# Finnish Version of the Specialist Outcomes and Barriers Analysis Scale: Evaluation of Psychometric Properties

**DOI:** 10.1097/NUR.0000000000000779

**Published:** 2023-10-06

**Authors:** Mea Mirella Marjatta Wright, Tarja Anneli Kvist, Santtu Juhani Mikkonen, Krista Susanna Jokiniemi

**Affiliations:** **Author Affiliations:** PhD student (Ms Wright), Professor (Dr Kvist), University lecturer (Dr Jokiniemi), Department of Nursing Science, Faculty of Health Sciences; and Research Manager (Dr Mikkonen), Department of Technical Physics, Faculty of Science and Forestry, University of Eastern Finland, Kuopio.

**Keywords:** advanced practice nursing, clinical nurse specialist, nurses, principal component analysis, psychometrics, validation

## Abstract

**Purpose:**

To evaluate the psychometric properties of the Finnish version of the Specialist Outcomes and Barriers Analysis Scale.

**Design:**

This was a cross-sectional survey study.

**Methods:**

Cultural adaptation of the translation and content validity of the translated instrument were assessed by expert panelists (n = 5) using the content validity index. The construct validity was assessed with principal component analysis using the survey data of Finnish registered nurses (n = 60). Scale reliability was assessed with Cronbach's *α* values. All study phases were conducted in 2021.

**Results:**

The items (n = 59) of the scale were critically evaluated by the experts. The full-scale content validity was revealed as excellent (0.92). In terms of construct validity, the scale was analyzed separately for outcomes and barriers. The outcomes section revealed a 5-component structure with an overall Cronbach's *α* coefficient of .96, and the barriers section, a 2-component structure with an overall Cronbach's *α* coefficient of .82, indicating adequate reliability of the scale.

**Conclusion:**

The Finnish version of the scale showed excellent content and construct validity. The Cronbach's *α* values represented adequate reliability of the Specialist Outcomes and Barriers Analysis scale when measuring nurses' perceived practice outcomes and barriers in the Finnish context.

Globally, there is an increasing demand for chronic disease management and prevention, which could be answered with the deployment of advanced practice nursing roles. The advanced practice nurse (APN) roles were introduced in the United Kingdom and Australia in the late 1980s,^1,2^ and around 2000, the APN roles emerged in the Nordic countries.^3^ Advanced practice nursing is one of the fastest-growing health professions in the United States,^4^ and several European countries are now implementing APN roles.^5^ The level of advanced practice autonomy is regulated by country policies and laws.^1^ A clinical nurse specialist (CNS) is an advanced practice registered nurse (RN) with graduate preparation (master's or doctoral degree) in nursing science.^1,6^ Clinical nurse specialists have extensive nursing knowledge, skills, and clinical expertise in a specialty area^7^ and can work in every field of healthcare.^1^ The CNS role has been developing in the United States and Canada for more than 60 years.^6,8^

The role of CNS may meet the unmet needs of diverse healthcare settings, such as the rising health expenditure due to chronic illnesses, as changes in the population and healthcare structures occur.^1^ Systematic literature reviews and intervention studies have found several beneficial outcomes of CNS-provided care. These include enhanced quality of life with lower complication rates,^9,10^ reduced length of stay and the number of readmissions in hospitals,^11,12^ support for early recovery,^13^ improved physical and psychological well-being of patients, improved quality of care and access to supportive care through collaborative management^10,11^ as well as improved patient satisfaction, and reduction in medication errors.^11^ However, a systematic review of controlled trials found inconsistent results of CNS-provided care, due to small sample sizes and failure of true randomization of patients.^14^ Thus, high-quality effectiveness research is still needed to reliably assess CNS-sensitive outcomes.

Finnish healthcare is based on public services to which everyone residing in the country is entitled. Healthcare professionals in Finland are divided into licensed professionals and professionals with a protected occupational title.^15^ The nurses' career pathway in Finland is presented in Figure 1. Universities of applied sciences (UASs) educate bachelor-level RNs, including midwives, paramedics, and public health nurses. After the bachelor-level education, nurses can acquire additional education in a specified field to become a specialized nurse (SN). To become SN, 30 to 60 European credit transfer system (ECTS) credits of further education are needed and provided by the UASs on different topics, such as wound care.^17^ Education preparing for APN roles can be studied in universities and UASs, for example, at the University of Åbo Akademi (2020),^18^ University of Oulu (2022),^19^ Laurea UAS, and Oulu UAS.^20,21^ The minimum education for the CNS role in Finland is a master's degree, which is worth between 90 and 120 ECTS credits.^22^ The exact number of SNs and APNs in Finland is unknown. The number of CNSs was estimated to be more than 100 in 2020.^23^

**FIGURE 1.**
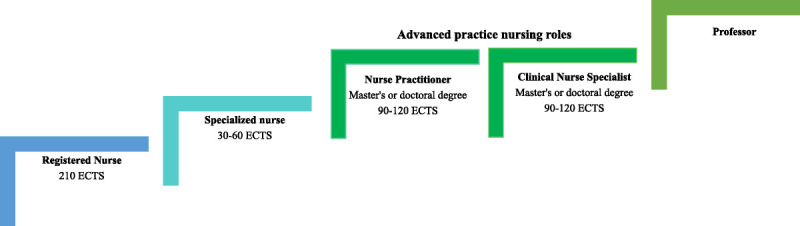
Nurses' career pathway in Finland (see, eg, FNA^16^).

Despite the 2-decade APN role development and initial national APN career model,^24^ APN education and continuous education are still in their early stages. To respond to the lack of continuous education and to meet the current challenges in healthcare, a 40-ECTS-credit continuing education (CE) program focusing on the core competencies of a CNS (the EFFICACY [Accessibility, Quality, and Safety for Health Services: Clinical Nurse Specialist Education] project) was developed and piloted at the University of Eastern Finland (UEF). The effectiveness of the EFFICACY project is measured with several reliable instruments. To assess the project's effectiveness on nurses' perceived practice outcomes and barriers, the Specialist Outcomes and Barriers Analysis (SOBA) Scale^25^ was translated into Finnish, and its content and construct validity was assessed. Instrument validation is reported in this study.

## THE EFFICACY PROJECT

**FIGURE 2.**
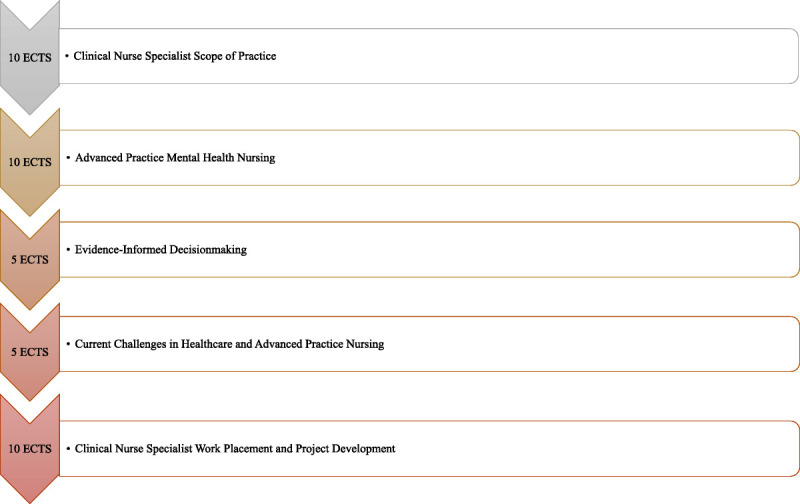
The structure of the EFFICACY curriculum (September 2021 to May 2023). Abbreviation: EFFICACY, Accessibility, Quality, and Safety for Health Services: Clinical Nurse Specialist Education.

The EFFICACY project is a CE project coordinated by the UEF, Department of Nursing Science (UEF, 2021), and funded by the European Social Fund. This project aimed to develop and implement a modern CE program for nurses to enhance competencies related to the scope of CNS practice. The program curriculum was developed by cross-tabulating international CNS curricula, recognizing emergent themes, and discussions with university lecturers. The CE program is a 2-year curriculum worth 40 ECTS credits. Thirty-five nurses from the Northern Savo area in Finland were accepted and started their studies in the fall of 2021. The CE program consists of 5 main topics, which are presented in Figure 2. The EFFICACY program is piloted between September 2021 and May 2023 (UEF, 2021).^26^ The EFFICACY project does not lead to a specific degree but amounts to CE credits. The translated and validated SOBA instrument will be used in assessing the development of nurses' perceived practice outcomes and barriers to their practice during the 2-year EFFICACY program among the program students and a control group. To our knowledge, no other instrument except SOBA exists to specifically measure nursing outcomes and barriers to practice related to the scope of CNSs.

## SPECIALIST OUTCOMES AND BARRIERS ANALYSIS SCALE

The SOBA instrument was first developed in 1994 by Smith and Waltman^25^ and presented in their study “Oncology Clinical Nurse Specialists' Perceptions of Their Influence on Patient Outcomes.” The instrument has since been used in studies conducted by Mayo et al^27^ and Kilpatrick et al.^7^ Smith and Waltman^25^ have analyzed the content and construct of the SOBA instrument in pilot studies. Principal factor solution with varimax rotation revealed adequate validity with Cronbach's *α* coefficient values from .73 to .83 between subscales. Principal component analysis (PCA) with varimax rotation was used by Mayo et al,^27^ revealing an 8-factor structure of the outcomes section that explained 68% of the variance and a 4-factor structure of the barriers section that explained 66% of the variance. The Cronbach's *α* coefficient values were .95 for the outcomes section and .84 for the barriers section of the scale, indicating excellent psychometric properties.

To our knowledge, this is the first study to address the content and construct validity of the SOBA instrument in a European context. Validating the instrument enables measurement of nurses' perception of their influence on patient outcomes in Finland and makes it possible to specifically monitor the perceived impact of different interventions for CNS-sensitive patient outcomes.

## METHODS

### Aim

The aim of this study was to evaluate the psychometric properties of the Finnish version of the SOBA instrument in a sample comprising expert panelists (n = 5) and Finnish RNs (n = 60).

### Design

The study was conducted in 2 phases: (1) a content validation study with expert panelists (n = 5) using the content validity index (CVI)^28,29^ and (2) a construct validation study with Finnish nurses (n = 60) using PCA.^30^ This study followed the STROBE (Strengthening the Reporting of Observational Studies in Epidemiology) statement.^31^ For further information, see Supplementary File 1, http://links.lww.com/NUR/A50.

#### Content Validation Study

The research objective was to examine the content validity and the cultural adaptation of the Finnish version of the SOBA instrument.

#### Construct Validation Study

The research objective was to evaluate the psychometric properties of the Finnish version of the SOBA instrument. The scale reliability was assessed with Cronbach's *α* values.

### Instrument

The SOBA instrument is divided into 2 sections: outcomes and barriers. The outcomes section contains 45 closed questions and 1 open-ended question. All the closed items use a Likert scale varying from 1 to 4: 1 = seldom influence this outcome (0-6 times a year), 2 = occasionally influence this outcome (7-11 times a year), 3 = frequently influence this outcome (1-3 times per month), and 4 = constantly influencing this outcome (at least once a week). The barriers section contains 14 closed items with a Likert scale ranging from 1 to 4: 1 = seldom encountered as a barrier (0-6 times a year), 2 = occasionally encountered as a barrier (7-11 times a year), 3 = frequently encountered as a barrier (1-3 times per month), and 4 = constantly encountered as a barrier (at least once a week). The original version of the instrument also contains a demographic section, which includes 12 questions about the respondent's background (eg, age, gender, and highest level of education).

The instrument was translated into Finnish in spring 2021. The following strategies were used to improve the quality of the translation: (1) translation and back-translation were performed by a certified translation service; (2) the translation was reviewed by an expert panelist (n = 5) and the EFFICACY project personnel; and (3) the original version and the translated version were compared with maintain conceptual equivalences. The wording and cultural adaptation of the translated items were evaluated by the expert panelists and the EFFICACY project personnel. Instead of the original English demographic section, we formulated our own background questions.

### Participants and Data Collection

#### Content Validation Study

The data were collected in August 2021 from purposefully selected expert panelists (n = 5) who were advanced practice nursing experts. Data were collected by a web-based survey, in which the participants were asked to rate each item based on a 4-point Likert scale (0 = irrelevant, 1 = slightly irrelevant, 2 = relevant, 3 = extremely relevant) on its relevance to the Finnish healthcare system and its cultural adaptation and wording.

#### Construct Validation Study

The data were collected from Finnish nurses attending the EFFICACY project CE program (n = 34) and a control group (n = 34) consisting of nurses with similar background working in the same organizations as the intervention group. The control group was handpicked by the EFFICACY project collaborators working in the affiliated organizations. The participants are working in university hospital settings, psychiatric inpatient and outpatient care, and somatic inpatient and polyclinic care in the Northern Savo area. Data were collected before the program in September 2021 by a self-administered online survey.

### Data Analysis

#### Content Validation Study

From the expert panelist ratings (n = 5), an item-level CVI (I-CVI) was computed by summing the number of panelists giving a rating of “relevant” or “extremely relevant” for each item divided by the total number of panelists. The I-CVI values ranged from 0 to 1. With fewer than 6 raters, the I-ICV is considered acceptable if the value is 1. The scale-level CVI with average calculation method (S-CVI/Ave) was measured by calculating the sum of the I-CVIs and dividing it by the total number of items. An S-CVI/Ave is considered acceptable with a value of 0.90 or higher.^29^

#### Construct Validation Study

The construct validity of the Finnish translation of the SOBA instrument was assessed with a cross-sectional survey study (n = 60). The PCA method was used, and the data were analyzed using the Statistical Package for the Social Sciences (version 27.0, 2017; IBM Corp, Armonk, New York). In total, 44 items were analyzed. The goals of PCA are to (1) extract the most important information from the data, (2) reduce the dimension of the data, (3) simplify the data set, and (4) analyze the structure of the observation and the variables. To achieve this, PCA formulates new variables: principal components.^30^ To get more interpretable components, oblique promax rotation was applied on the outcomes scale and orthogonal varimax rotation on the barriers scale.^31^ Missing values were not imputed (n = 43). Scree plots were used to determine the number of components to extract.

### Ethical Issues

The research plan of the broader study considering the EFFICACY program has been evaluated by the UEF on Research Ethics (statement no. 10/2021). Research permits were obtained from the participating organizations as a part of the EFFICACY program. The study was conducted based on ethical principles of the Finnish National Board on Research Integrity.^33^ Informed consent was obtained from the respondents. Participation was voluntary, and the respondents were able to drop out at any point of the study. This study also followed general European Union data privacy policy (EU 2016/679) and the Finnish Data Privacy Act (2018/1050).

## RESULTS

### Content Validation Study

Of the 5 expert panelists, 4 were women, 3 had a master's degree in health sciences, and the rest had a PhD in health sciences. The mean age of the expert panelists was 43, and they worked as CNSs (n = 3) or scholars with experience in instrument development and APN roles (n = 2).

The expert panelists and the EFFICACY project personnel evaluated the translation in a Finnish context, and the translation maintained idiomatic, conceptual, and experiential equivalences. I-CVI values ranged between 0.4 and 1.0. A total of 16 items scored an I-CVI value under 1 (Table 1). After careful consideration by the EFFICACY personnel, items were left in the Finnish translation after the content validity study. The S-CVI/Ave for the whole scale was 0.92.

**Table 1:** Items Scoring Under the Preferred I-CVI Value

Item	I-CVI
Cost of stay	0.4
Cost of treatment	0.6
Complication rate	0.8
Prevention of readmission	0.8
Correct medical diagnosis	0.4
Correct medical treatment	0.6
Correct nursing diagnosis	0.8
Earlier start of treatment	0.8
Compliance rate	0.8
Control of treatment adverse effects	0.8
Reintegration of patient to school, work, or community	0.8
Consultation—caregiver	0.8
Referrals to resources	0.6
Staff nurse resistance	0.8
Difficult physician personality^a^	0.4
Lack of secretarial support	0.8

Abbreviation: I-CVI, item content validity index.^28^

^a^Omitted from the scale because of low CVI value and theoretical reasoning.

#### Construct Validation Study

Twenty-six nurses attending the EFFICACY program and 34 nurses from the control group answered the questionnaire, with a response rate of 87%. The intervention and control groups had comparable demographic and clinical characteristics. Within both groups, the majority of the respondents were women, with a mean age of 44 years. Most of the respondents were titled as RNs in both groups. The demographic information of the participants is presented in Table 2.

**Table 2:** Demographic Information of the Construct Validity Study Participants (n = 60)

	Intervention (n = 35)	Control (n = 34)
Response rate	76.5% (n = 26)	100% (n = 34)
Gender^a^	Female = 84.6% (n = 22) Male = 11.5% (n = 3)	Female = 85.7% (n = 30) Male = 11.4% (n = 4)
Mean age, y	43.6 (min, 28; max, 61; SD, 8.3)	44.5 (min, 31; max, 59; SD, 7.3)
Operational level	Unit = 92.3% (n = 24) Division = 3.8% (n = 1) Organization = 3.8% (n = 1)	Unit = 85.7% (n = 30) Division = 5.7% (n = 2) Other = 5.7% (n = 2)
Highest level of education^b^	RN = 85.6% (n = 22) Master's degree, UAS = 7.7% (n = 2) Master's degree, university = 7.7% (n = 2)	RN = 80% (n = 28) Master's degree, UAS = 8.6% (n = 3) Master's degree, university = 8.6% (n = 3)
Profession^b^	Registered nurse = 76.9% (n = 20) Midwife = 3.8% (n = 1) Nurse manager = 11.5% (n = 3) Other = 7.7% (n = 2)	Registered nurse = 74.3% (n = 26) CNS or specialist nurse = 2.9% (n = 1) Nurse manager = 17.1% (n = 6) Other = 2.9% (n = 1)
Years in the current profession, mean	10.5 (min, 1; max, 27; SD, 7.3)	12.3 (min, 1; max, 31; SD, 8.3)
Years in the current unit, mean	7.1 (min, 0; max, 25; SD, 7.1)	6 (min, 0; max, 24; SD, 6.2)
Years in the current organization, mean	11.3 (min, 2; max, 20; SD, 8.6)	13.5 (min, 0; max, 30; SD, 7.8)

Abbreviations: CNS, clinical nurse specialist; RN, registered nurse; UAS, university of applied sciences.

^a^One missing answer in the intervention and control groups.

^b^One missing answer in the control group.

#### Outcomes Section of the Scale

Forty-four items were analyzed in the outcomes section. The outcomes section revealed 5 principal components, accounting for 67.6% of the total variance. With our data, the Cronbach's *α* was .958 for the whole outcomes scale. The item loadings varied between 0.446 and 0.916, and all item loadings were greater than 0.3, which is considered acceptable.^34^

When the original factor structures (determined by Smith and Waltman^25^ in 1994, represented in Table 3 with letters A-E) were used, the Cronbach's *α* values were .912 for “patient and family response to care,” .836 for “cost of care,” .950 for “organizational processes,” .842 for “consultative/interdisciplinary processes,” and .914 for “research processes.” The items loaded onto the original factors, with few modifications (Table 3).

**Table 3:** Principal Component Analysis Item Loadings With Promax Rotation and Kaiser Normalization (n = 60)

Item	Component					Cronbach's *α* if Item Deleted
	1	2	3	4	5	
D6 factors/processes—referrals to resources	**0.903**	−0.012	−0.067	0.001	0.080	.957
C13 factors/processes—involvement in quality assurance	**0.859**	−0.110	−0.180	0.108	0.107	.957
C7 factors / processes—nurse retention	**0.796**	0.016	0.100	0.136	−0.123	.956
C6 factors/processes—consultation-administration	**0.795**	0.091	−0.119	−0.231	0.260	.957
C9 factors/processes—staff productivity	**0.790**	−0.015	0.065	0.020	0.105	.956
C8 factors/processes—nurse satisfaction	**0.780**	0.003	0.156	−0.004	0.052	.956
C12 factors/processes—staff utilization of effective interventions	**0.766**	0.103	−0.029	−0.092	0.149	.957
C2 factors/processes—staff skill	**0.699**	−0.080	0.059	0.007	0.325	.956
D5 factors/processes—support group facilitation	**0.527**	0.230	0.303	0.058	−0.121	.956
C11 factors/processes—improved staff communication	**0.520**	−0.024	0.093	0.215	0.186	.956
C10 factors/processes—testing product effectiveness	**0.485**	−0.163	0.409	0.277	−0.114	.956
A6 patient outcome—prevention of injury^a^	**0.446**	0.231	−0.088	0.403	−0.199	.957
A8 patient outcome—patient/family anxiety level	0.195	**0.916**	−0.359	−0.080	−0.055	.958
A7 patient outcome—compliance rate	0.021	**0.874**	−0.095	−0.034	−0.013	.957
A5 patient outcome—improved coping for the family	0.172	**0.809**	0.058	−0.258	0.083	.957
A10 patient outcome—control of treatment adverse effects	0.036	**0.762**	0.174	0.075	−0.189	.957
A9 patient outcome—patient/family satisfaction	0.000	**0.743**	−0.194	0.210	−0.032	.958
A11 patient outcome—reintegration of patient to school, work, or community	−0.052	**0.740**	−0.070	−0.189	0.206	.958
A12 patient outcome—improved patient/family knowledge	−0.317	**0.694**	0.171	0.067	0.035	.958
A4 patient outcome—improved coping for patient	−0.168	**0.685**	−0.090	0.272	0.209	.957
D7 factors/processes—advocacy for patient/family with physician^b^	0.316	**0.531**	0.343	−0.168	−0.066	.956
A3 patient outcome—correct nursing diagnosis	0.031	**0.523**	0.165	0.247	0.188	.956
B8 patient outcome—earlier start of treatment	0.026	**0.497**	−0.023	0.127	0.361	.957
D3 factors/processes—multidisciplinary cooperation	−0.132	−0.206	**0.911**	0.095	0.108	.957
E1 factors/processes—conducting research	0.055	0.096	**0.895**	−0.270	−0.060	.957
D4 factors/processes—improved interdisciplinary communication	0.105	−0.076	**0.836**	−0.024	−0.103	.957
E2 factors/processes—dissemination of research findings	0.167	−0.188	**0.822**	0.067	−0.047	.957
E3 factors/processes—utilization of research findings	0.214	−0.021	**0.684**	−0.007	0.119	.956
B7 patient outcome—correct medical treatment^c^	−0.263	0.358	**0.502**	0.195	0.150	.957
B6 patient outcome—correct medical diagnosis^c^	−0.078	0.287	**0.466**	−0.066	0.296	.957
B4 patient outcome—complication rate	−0.142	−0.057	0.000	**0.788**	0.127	.958
B1 patient outcome—length of stay/timely discharge	−0.053	0.208	−0.106	**0.762**	−0.050	.958
B9 patient outcome—appropriate care products	0.218	0.167	0.070	**0.703**	−0.285	.957
B2 patient outcome—cost of stay	0.190	−0.340	0.013	**0.679**	0.137	.958
B3 patient outcome—cost of treatment	0.097	−0.266	−0.030	**0.551**	0.448	.957
B5 patient outcome—prevention of readmission	−0.136	0.305	0.015	**0.525**	0.279	.957
A1 patient outcome—comfort level	0.167	0.362	−0.095	**0.493**	−0.124	.957
C3 factors/processes—program development	0.541	−0.097	−0.147	−0.023	**0.710**	.957
D2 factors/processes—consultation-caregiver	0.055	0.289	−0.010	0.088	**0.675**	.956
C4 factors/processes—procedure development	0.483	−0.062	−0.102	0.018	**0.675**	.956
C5 factors/processes—improved documentation	0.211	0.125	0.234	−0.153	**0.555**	.957
D1 factors/processes—consultation - physician	0.072	0.439	0.075	−0.174	**0.537**	.956
C1 factors/processes—staff knowledge	0.431	−0.128	0.221	−0.021	**0.464**	.956
A2 patient outcome—self-care ability^c^	−0.147	0.389	−0.057	0.352	**0.455**	.957

Abbreviations: A, patient and family response to care; B, cost of care; C, organizational processes; D, consultative/interdisciplinary processes; E, research processes.

^a^Despite the loading, item placed in component 4.

^b^Despite the loading, item placed in component 3.

^c^Despite the loading, item placed in component 2.

Based on theoretical reasoning (Table 3), the item “A6 patient outcome—prevention of injury” was placed in component 4 despite its loading; item “D7 factors/processes—advocacy for patient/family with physician” was placed in component 3; and items “B7 patient outcome—correct medical treatment,” “B6 patient outcome—correct medical diagnosis,” and “A2 patient outcome—self-care ability” were placed in component 2. After these alterations, the Cronbach's *α* was .921 for “patient and family response to care,” .860 for “cost of care,” .946 for “organizational processes,” .901 for “consultative/interdisciplinary processes,” and .906 for “research processes.” Other items loading onto different components than the original factor structure were left in the components with the greatest loadings due to theoretical reasoning (Table 3).

#### Barriers Section of the Scale

Fifteen items were analyzed in the barriers section. Two items with the lowest communalities (lack of personal expertise = 0.213 and patient/family resistance = 0.151) were omitted from the scale. In addition to these, the item with the lowest CVI value (difficult physician personality, CVI = 0.4) was deleted. The remaining 12 items revealed 2 principal components, accounting for 50.6% of the total variance. The components were named as (1) organizational and interpersonal barriers and (2) workflow-related barriers. The item loadings varied between 0.501 and 0.815 (Table 4). With our data, the Cronbach's *α* was .821 for the whole barriers scale. When the given component structure was used (Table 4), the Cronbach's *α* values were .814 for component 1 and .725 for component 2.

**Table 4:** Principal Component Analysis Item Loadings With Varimax Rotation and Kaiser Normalization (n = 60)

	Component		
	1	2	Cronbach's *α* if Item Deleted
Internal regulations (ie, institutional or unit policies or procedures)	**0.825**	0.136	.821
Lack of peer support	**0.715**	0.225	.822
Physician resistance	**0.689**	−0.311	.845
Staff nurse resistance	**0.655**	0.261	.823
Lack of administrative support	**0.645**	0.418	.814
Lack of personnel	**0.545**	0.526	.814
Inadequate space	**0.459**	0.338	.829
Lack of time	−0.011	**0.888**	.826
Multiple job expectations	0.125	**0.670**	.831
Lack of funding	0.099	**0.625**	.834
Lack of secretarial support	0.274	**0.577**	.826
Inconvenient layout of office and/or work areas	0.272	**0.506**	.831

## DISCUSSION

This study aimed to evaluate the psychometric properties of the Finnish version of the SOBA instrument^25^ in a sample of Finnish RNs. The instrument has previously been used in the United States and Canada with excellent psychometric properties.^7,24,25^ The content validity of the Finnish version was satisfactory, with 16 items scoring under the preferred value. This may be partly due to strict I-CVI requirements with 5 expert panelists and partly due to the differences between the healthcare systems of Finland and the United States. Finnish healthcare is based on public services: everyone residing in Finland is entitled to healthcare, and private healthcare is only an addition to public services.^15^ Healthcare in the United States consists of several systems that serve different segments of the population, with the majority of the population covering their healthcare with private health insurance.^35^ After careful consideration, 13 items scoring under the preferred I-CVI value were left in the translated scale, so that the results would be as comparable to previous studies using this instrument as possible. The content validity of the whole scale was 0.92, indicating acceptable content validity.

In contrast to Mayo and colleagues'^25^ study with 8 factors, items in our study loaded onto original factors defined by Smith and Waltman,^25^ with few alterations. In the outcomes section, 5 items were placed onto different components after theory-based consideration (Table 4). The differences between the Finnish and US healthcare systems were considered at this point. Finnish healthcare is divided into primary care and specialized care, with the latter usually offered at district hospitals and the former at healthcare centers.^15^ There was no previous factor structure provided for the barriers section of the scale, so the components were formed and named with the highest item loadings (Table 4).

The scale's reliability was assessed with Cronbach's *α* values consistent with previous studies. The Cronbach's *α* coefficients varied from .73 to .83 between subscales in the original instrument development study by Smith and Waltman.^25^ Similar to our findings, the outcomes section Cronbach's *α* coefficients were .95 and .84 in the barriers section in the study conducted by Mayo et al.^27^ The scale reliability was assessed with Cronbach's *α* values, which were high throughout the subscales.

The results of this study can be utilized in the wider advanced practice nursing community. Previous research has indicated a connection between nurses' job satisfaction, nurse retention, and patient outcomes.^36^ Insufficient work experience and training possibilities are also connected to nurse turnover and indicated in research.^37,38^ The SOBA instrument was found to demonstrate excellent content and construct validity, which is in line with previous research.^7,25,27^ The instrument enables measurement of nurses' perceived practice outcomes and barriers to practice in the Finnish context. Future research should examine the possible connection of perceived practice outcomes and job satisfaction in finding new solutions to the nursing shortage by increasing the attraction of the healthcare professions.

### Strengths and Limitations

Multiple methods were undertaken to increase the strength of this study. In the translation phase, we used certified translation services and a pilot study of 5 expert panelists to ascertain the soundness of the 2-step Finnish translation of the SOBA instrument.^28,29^ To increase the reliability of reporting the results, this study adhered to the STROBE checklist (Supplementary File 1, http://links.lww.com/NUR/A50). However, some limitations should be mentioned. During the translation process, one of the scale items (advocacy for patient/family with departments) was omitted from the Finnish version of the scale by mistake and was left out of the scale. Furthermore, the use of 6 or more expert panelists would have probably increased the reliability of the content validation study: 6 panelists were asked, but only 5 agreed to participate. The sample size in our study is rather small, and the convenience sampling may limit the generalizability of our findings.

Although the positive effects of advanced practice nursing roles are indicated in research, these roles are still developing in the Nordic countries. Because there are still very few of these roles (>400 NPs, >100 CNSs) in Finland,^24^ the respondents in this study were RNs. The respondents are, however, attending a CE program aimed to enhance CNS competencies. We chose this instrument because it will be used to measure the effectiveness and the growth of these competencies among the EFFICACY project students, who were mostly RNs at the entry level of the program. The SOBA instrument has been developed and previously used to measure CNS outcomes and barriers, and this may also limit the generalizability of our findings. The study findings should be replicated with a larger sample and different population of nurses in the future.

## CONCLUSION

A 2-phase study was conducted to evaluate the psychometric properties of the Finnish version of the SOBA Scale. The SOBA instrument has not been widely used since its development in 1994 despite research finding excellent psychometric properties. We found evidence of validity and reliability for the Finnish version of the SOBA instrument in a sample comprising Finnish RNs. In total, 3 items were omitted from the Finnish version of the scale. The SOBA Scale may be used to examine nurses' perceived practice outcomes and barriers and to support the evaluation of effective nursing practice within healthcare systems. This study also strengthens the reliability of the SOBA Scale, which has now been found to have acceptable psychometric properties in 3 languages (English, French, Finnish) in studies conducted in 3 different countries (United States, Canada, Finland).^7,25,27^

Principal component analysis is a good tool for validating this type of scale and can specifically be applied on data of smaller sample sizes. If the number of observations were higher, confirmatory factor analysis could be used to confirm the results. This could be a task for a subsequent study.

### Relevance to Clinical Practice

To our knowledge, there has previously been no instrument in the Finnish language to measure SNs' perceived practice outcomes and barriers. A translated and validated SOBA Scale will enable standardized measurement of perceived nursing practice outcomes and barriers in Finland. This study also strengthens the reliability of the SOBA Scale developed in the United States in 1994.

## Supplementary Material
